# Sedentary behavior and residual-specific mortality

**DOI:** 10.15171/hpp.2016.32

**Published:** 2016-10-01

**Authors:** Paul D. Loprinzi, Meghan K. Edwards, Eveleen Sng, Ovuokerie Addoh

**Affiliations:** ^1^Jackson Heart Study Vanguard Center of Oxford, Center for Health Behavior Research, Physical Activity Epidemiology Laboratory, Department of Health, Exercise Science and Recreation Management, The University of Mississippi, University, MS 38677, USA; ^2^Center for Health Behavior Research, Physical Activity Epidemiology Laboratory, Department of Health, Exercise Science and Recreation Management, The University of Mississippi, University, MS 38677, USA; ^3^Mississippi Baptist Health Systems – Baptist Outpatient Cardiac Rehabilitation, Center for Health Behavior Research, Physical Activity Epidemiology Laboratory, Department of Health, Exercise Science and Recreation Management, The University of Mississippi, University, MS 38677, USA

**Keywords:** Accelerometry, Epidemiology, Physical activity, Survival

## Abstract

**Background: ** The purpose of this study was to examine the association of accelerometer-assessed sedentary behavior and residual-specific mortality.

**Methods:** Data from the 2003-2006 National Health and Nutrition Examination Survey (NHANES) were used (N = 5536), with follow-up through 2011. Sedentary behavior was objectively measured over 7 days via accelerometry.

**Results:** When expressing sedentary behavior as a 60 min/day increase, the hazard ratio across the models ranged from 1.07-1.40 (P < 0.05). There was evidence of an interaction effect between sedentary behavior and total physical activity on residual-specific mortality (Hazard ratio_interaction_ [HR] = 0.9989; 95% CI: 0.9982-0.9997; P = 0.008).

**Conclusion:** Sedentary behavior was independently associated with residual-specific mortality. However, there was evidence to suggest that residual-specific mortality risk was a function of sedentary behavior and total physical activity. These findings highlight the need for future work to not only examine the association between sedentary behavior and health independent of total physical activity, but evaluate whether there is a joint effect of these two parameters on health.

## Introduction

Regular participation in physical activity is irrefutably beneficial in improving physical and psychological health. The National Physical Activity Plan (http://www.physicalactivityplan.org/) highlights the importance of regular physical activity across the lifespan and identifies evidence-based physical activity promotion strategies across numerous health sectors, including public health. Emerging research demonstrates that sedentary behavior, independent of physical activity, is associated with increased risk of all-cause mortality,^1-3^ coronary artery disease (CAD)-specific mortality,^1-3^ coronary heart disease-specific mortality^1^ and cancer-specific mortality.^1,2^ Notably, only one study, to date, has evaluated the association of physical activity and residual-specific mortality,^4^ with this study demonstrating a significant, inverse association between physical activity and residual-specific mortality. Identical to the only other study on this topic,^4^ residual-specific mortality is defined herein as all other causes of death aside from the first 9 major causes of death identified,^5^ including (1) diseases of the heart; (2) malignant neoplasms; (3) chronic lower respiratory diseases; (4) accidents (unintentional injuries); (5) cerebrovascular diseases; (6) Alzheimer disease; (7) diabetes mellitus; (8) influenza and pneumonia; and (9) nephritis, nephrotic syndrome and nephrosis. Examining the effect of sedentary behavior on residual-specific mortality is noteworthy of investigation, given the large proportion of deaths not attributed to the first 9 major causes of death defined in the recoded International Classification of Diseases, 10th edition (ICD-10).^6^ For example, as shown in the first paragraph of the results section of this manuscript, the highest proportion of deaths (32%) observed within our study were attributed to residual-specific mortalities. Thus, the purpose of this study was to examine the association between sedentary behavior and residual-specific mortality. We hypothesized that, independent of physical activity, sedentary behavior would be positively associated with residual-specific mortality.

## Materials and Methods

### Design and participants

Data from the 2003-2006 National Health and Nutrition Examination Survey (NHANES) were used. Data from participants in these cycles were linked to death certificate data from the National Death Index. Person-months of follow-up were calculated from the date of the interview until date of death or censoring on December 31, 2011, whichever came first. Analyses are based on data from 5536 adults (20-85 years) who had data on the study variables.

The NHANES is an ongoing survey conducted by the Centers for Disease Control and Prevention (CDC) that uses a representative sample of non-institutionalized US civilians selected by a complex, multistage, stratified, clustered probability design. The multistage design consists of 4 stages, including the identification of counties, segments (city blocks), random selection of households within the segments, and random selection of individuals within the households. Procedures were approved by the National Center for Health Statistics review board. Consent was obtained from all participants prior to data collection. Further information on NHANES methodology and data collection is available on the NHANES website (http://www.cdc.gov/nchs/nhanes.htm).

### Measurement of sedentary behavior

Sedentary behavior was assessed using the ActiGraph 7164 accelerometer. SAS (version 9.2) was used to reduce accelerometry data to those with ≥4 days of ≥10 h/day of monitored data and integrate it into 1 minute time intervals. Nonwear time was identified as ≥60 consecutive minutes of zero activity counts, with allowance for 1-2 minutes of activity counts between 0 and 100. Sedentary behavior was defined as counts/min <99.^7^ Moderate-to-vigorous physical activity (MVPA) and light-intensity physical activity were included as covariates in the model. The Troiano cut-point (2020 counts/min) was used to determine time spent in MVPA.^8^ Light-intensity physical activity was defined as counts/min of 100-2019.

### Statistical analysis

Statistical analyses were performed via procedures from survey data using Stata (v.12; College Station, TX, USA); statistical significance was set at an alpha of 0.05. Analyses accounted for the complex survey design employed in NHANES by utilizing sample weights, primary sampling units and strata via the Taylor series (linearization) method.

Weighted Cox proportional hazard models were used to examine the association between sedentary behavior and residual-specific mortality; Harrell’s C concordance statistic and the proportional hazard assumption were evaluated for each Cox proportional model. As shown in Table 1, 7 primary models were evaluated, which included:

Model 1 – Unadjusted;

Model 2- Adjusted for age, gender, and race-ethnicity;

Model 3 - Adjusted for age, gender, race-ethnicity and weight status;

Model 4 - Adjusted for age, gender, race-ethnicity weight status, mean arterial pressure, high-density lipoprotein cholesterol (HDL-C), total cholesterol, C-reactive protein (CRP), physical activity comparison to the last year, congestive heart failure (CHF), CAD, heart attack, stroke, emphysema, chronic bronchitis, diabetes, smoking status and cancer;

Model 5 – Same as Model 4 but also included MVPA;

Model 6 – Same as Model 5, but instead of including MVPA as a covariate, light-to-vigorous physical activity (i.e., total physical activity) was included; and

Model 7 – Same as Model 6, but sedentary behavior was now expressed relative to the accelerometer wear time, i.e., ([min/day of sedentary behavior / min/day of accelerometer wear time]*100)

In addition to these 7 primary models, and as shown in Table 1, additional models were evaluated, including those who did not die within the first 12 and 24 months, participants 50+ years of age, and participants without CHF, CAD, heart attack, stroke, emphysema, chronic bronchitis, diabetes, and cancer. Further, multiplicative interaction models were computed by creating a cross-product term, and including this cross-product term, along with the main effects and the covariates, in a Cox proportional hazard model.

With regard to the covariates, age, gender, race-ethnicity, changes in physical activity, and smoking status were assessed via a self-report questionnaire. With regard to changes in physical activity, participants were asked, “*How does the amount of activity that you reported for the past 30 days compare with your physical activity for the past 12 months*? (response options: more active, less active or about the same.” Regarding the chronic diseases (CHF, CAD, heart attack, emphysema, chronic bronchitis, stroke, and diabetes), participants were asked if they had ever been told by a physician or other health professional that they had this disease. In addition to diabetes being assessed via self-report of physician diagnosis, here we also defined individuals as having diabetes if they had a fasting plasma glucose ≥ 126 mg/dL or an A1C (glycated hemoglobin) ≥ 6.5%. Using the average of up to four manually assessed blood pressure measurements, mean arterial pressure was calculated using the following formula: ([(diastolic blood pressure × 2) + systolic blood pressure]/3). HDL-C and total cholesterol were assessed enzymatically in serum or plasma via a blood sample. High sensitivity CRP concentration was quantified using latex-enhanced nephelometry. Lastly, using measured height and weight to calculate body mass index, weighted status was defined as normal weight (18.5-24.9 kg/m^2^), overweight (25.0-29.9 kg/m^2^), or obese (30+ kg/m^2^); notably, those with a body mass index <18.5 were excluded from analysis.

## Results

Among the analyzed sample of 5536 participants, 510 deaths accrued over the follow-up period. Among these 510 deaths, 112 were from diseases of the heart; 140 from malignant neoplasms; 26 from chronic lower respiratory diseases; 12 from accidents (unintentional injuries); 29 from cerebrovascular diseases; 13 from Alzheimer disease; 15 from diabetes; 9 from influenza or pneumonia; 10 from nephritis, nephrotic syndrome or nephrosis; and 144 from all other causes (residual). The outcome of interest in this study was the 144 residual-specific mortalities. Notably, the exact causes of these residual mortalities is not available in the publically accessible NHANES linked mortality data.

Table 2 displays the weighted characteristics of the study variables. Compared to those alive at censor, those who were decreased had a higher sedentary behavior level at baseline, engaged in less physical activity, were older, had a higher CRP level, and were more likely to have the evaluated chronic diseases. When compared to those with a below-median sedentary behavior level, those with above-median sedentary behavior had a higher residual-specific mortality rate, engaged in less physical activity, were older, and were more likely to have the evaluated chronic diseases.

Table 1 displays the weighted Cox proportional hazard results. Results were consistent across all models. Participants with a higher sedentary behavior level at baseline had an increased hazard for residual-specific mortality. When expressing sedentary behavior as a 60 min/day increase, the hazard ratio across the models ranged from 1.07-1.40, suggesting a 7%-40% increased hazard of residual-specific mortality for every 60 min/day increase in sedentary behavior. With the exception of the unadjusted model, the Harrell’s C concordance statistic were reasonable (C > 0.70) for all models. The proportional hazards assumption was not violated for any of the models (*P* > 0.05 for all).

There was no evidence of a multiplicative interaction effect of sedentary behavior and MVPA on residual-specific mortality (Hazard ratio_interaction_ [HR] = 0.994; 95% CI: 0.988-1.001; *P* = 0.08). However, the interaction term for sedentary behavior and total physical activity was significant (HR_interaction_ = 0.9989; 95% CI: 0.9982-0.9997; *P* = 0.008). When stratifying by the median level of total physical activity (≥359 min/day &< 359 min/day), and after complete adjustment (Model 4), sedentary behavior was not associated with residual-specific mortality among those above the median total physical activity level (HR = 1.09; 95% CI: 0.91-1.30; *P* = 0.32). However, sedentary behavior was associated with residual-specific mortality among those below the median total physical activity level (HR = 1.26; 95% CI: 1.11-1.41; *P* = 0.001). Among the 144 residual-specific deaths, 41 occurred among those above the median total physical activity level and 103 occurred among those below the median total physical activity level. Notably, there was no evidence of a multiplicative interaction effect for age and sedentary behavior on residual-specific mortality (HR = 1.00; 95% CI: 0.997-1.01; *P *= 0.18), nor for gender and sedentary behavior (HR = 0.99; 95% CI: 0.78-1.25; *P *= 0.96) or BMI and sedentary behavior (HR = 1.00; 95% CI: 0.98-1.03; *P* = 0.54).

**Table 1:** Weighted Cox proportional hazard results examining the association between sedentary behavior (expressed as a 60 min/day increase) and residual-specific mortality, 2003-2006 NHANES (N = 5536)

**60 Min/day increase in sedentary behavior**	** HR**	**95% CI**	***P*** ** value for HR**	**Harrell’s C concordance statistic**	***P*** ** value for proportional hazard assumption**
Model 1	1.40	1.29-1.51	<0.001	0.68	0.82
Model 2	1.21	1.12-1.31	<0.001	0.83	0.22
Model 3	1.21	1.12-1.31	<0.001	0.83	0.28
Model 4	1.21	1.10-1.32	<0.001	0.84	0.42
Model 5	1.18	1.06-1.29	0.002	0.85	0.50
Model 6	1.18	1.04-1.34	0.01	0.84	0.42
Model 7	1.07	1.01-1.14	0.03	0.84	0.41
Model 6 but among those who did not die in first 12 months of follow-up (N = 5491)	1.17	1.02-1.33	0.02	0.85	0.60
Model 6 but among those who did not die in first 24 months of follow-up (N = 5429)	1.16	1.00-1.35	0.05	0.86	0.20
Model 6 but among those not having CHF at baseline (N = 5349)	1.15	1.02-1.28	0.01	0.84	0.76
Model 6 but among those not having CAD at baseline (N = 5280)	1.17	1.03-1.32	0.01	0.85	0.27
Model 6 but among those not having heart attack at baseline (N = 5277)	1.19	1.06-1.34	0.004	0.85	0.35
Model 6 but among those not having stroke at baseline (N = 5340)	1.20	1.06-1.36	0.005	0.84	0.43
Model 6 but among those not having emphysema at baseline (N = 5425)	1.15	1.02-1.31	0.02	0.85	0.48
Model 6 but among those not having bronchitis at baseline (N = 5211)	1.17	1.05-1.31	0.007	0.85	0.20
Model 6 but among those not having diabetes at baseline (N = 4777)	1.19	1.05-1.35	0.01	0.86	0.52
Model 6 but among those not having cancer at baseline (N = 5016)	1.22	1.06-1.39	0.005	0.86	0.37
Model 6 but restricted to those 50+ yrs of age at baseline (N = 2895)	1.21	1.05-1.40	0.009	0.78	0.75

Abbreviations: CAD, coronary artery disease; HR, hazard ratio; CHF, congestive heart failure; NHANES, National Health and Nutrition Examination Survey.

**Table 2:** Weighted characteristics of the study variables, 2006-2003 NHANES (N=5536)

	**Point Estimate (95% CI)**				
**Variable**	**Entire sample** **(N = 5536)**	**No residual death** **(N = 5392)**	**Residual death** **(N = 144)**	**Below median sedentary** ^a^ **(N = 2760)**	**Above median sedentary** ^a^ **(N = 2771)**
Residual-specific mortality, n	144	0	144	41	103
Residual-specific mortality, %	1.7	-	-	0.9	2.4
Follow-up duration, mean months	80.6 (78.1-83.2)	81.3 (78.6-83.8)	45.3 (42.0-48.6)	81.8 (79.1-84.5)	79.4 (76.9-81.9)
PM of observation, mean	441197	434636	6561	224862	215918
Residual-specific mortality rate per 1000 PM of observation	0.32 (0.27-0.38)	-	21.9 (18.6-25.8)	0.18 (0.13-0.24)	0.47 (0.39-0.58)
Sedentary behavior, mean min/day	484.9 (481-488)	483.5 (480-486)	566.1 (543-588)	395.7 (393-397)	576.6 (573-579)
% of monitored time in sedentary behavior, mean %	56.5 (56.1-56.9)	56.4 (55.9-56.7)	66.5 (63.9-69.0)	48.3 (48.0-48.6)	65.0 (64.6-65.3)
MVPA, mean min/day	23.4 (22.3-24.5)	23.7 (22.5-24.8)	7.8 (5.3-10.3)	29.0 (27.6-30.5)	17.7 (16.4-18.9)
Light-to-vigorous, mean min/day	372.5 (368-376)	373.9 (369-378)	287.4 (259-315)	427.8 (422-432)	315.6 (312-319)
Accelerometer-wear time, mean h/day	14.3 (14.2-14.4)	14.3 (14.2-14.4)	14.2 (13.7-14.7)	13.7 (13.6-13.8)	14.87 (14.80-14.9)
Age, mean years	48.3 (47.4-49.2)	48.0 (47.1-48.9)	68.1 (65.1-71.0)	44.9 (43.8-46.0)	51.9 (50.6-53.1)
Female, %	50.7	50.8	49.0	51.3	50.1
Race-ethnicity, %					
Mexican American	7.8	7.9	4.2	11.1	4.4
Other Hispanic	3.3	3.3	2.3	4.4	2.2
Non-Hispanic White	74.5	74.5	78.7	71.0	78.2
Non-Hispanic Black	9.3	9.4	7.4	9.0	9.6
Other	5.0	5.0	7.4	4.5	5.6
Body mass index, mean kg/m^2^	28.4 (28.1-28.7)	28.4 (28.1-28.7)	28.2 (26.9-29.5)	28.0 (27.6-28.3)	28.8 (28.4-29.2)
Weight status, %					
Normal weight (18.5-24.9 kg/m^2^)	31.5	31.5	34.8	33.6	29.5
Overweight (25.0-29.9 kg/m^2^)	35.9	35.9	33.9	35.3	36.5
Obese (30+ kg/m^2^)	32.6	32.6	31.2	31.1	34.0
Mean arterial pressure, mean mm Hg	88.5 (87.9-89.0)	88.5 (87.9-89.0)	88.0 (85.1-90.9)	88.2 (87.6-88.8)	88.7 (88.1-89.3)
HDL-C, mean mg/dL	54.5 (54.0-55.1)	54.6 (54.0-55.1)	53.9 (50.6-57.1)	55.1 (54.2-55.8)	54.0 (53.2-54.8)
Total cholesterol, mean mg/dL	201.5 (200-203)	201.5 (200-202)	198.0 (184-211)	201.8 (199-204)	201.1 (199-203)
CRP, mean mg/dL	0.41 (0.38-0.43)	0.40 (0.37-0.43)	0.69 (0.31-1.05)	0.39 (0.34-0.43)	0.43 (0.39-0.46)
Activity comparison to last year, %					
More active	19.4	19.5	16.2	20.6	18.3
Less active	22.4	22.4	19.0	20.3	24.6
About the same	58.2	58.1	64.9	59.1	57.2
CHF, %	2.5	2.3	16.5	1.4	3.6
CAD, %	3.7	3.7	9.0	2.4	5.1
Heart attack, %	3.7	3.5	13.3	2.6	4.8
Stroke, %	2.5	2.4	8.7	1.7	3.4
Emphysema, %	1.8	1.7	8.5	1.5	2.1
Chronic bronchitis, %	6.5	6.3	17.5	5.7	7.3
Diabetes, %	10.2	10.0	21.9	7.5	12.9
Smoking status, %					
Smokes every day	17.7	17.8	14.0	19.3	16.1
Smokes most days	3.2	3.2	5.0	3.9	2.5
Former smoker	27.0	26.8	37.7	25.4	28.7
Never smoker	52.1	52.2	43.3	51.4	52.7
Cancer, %	8.9	8.8	17.3	6.7	11.2

Abbreviations: CAD, coronary artery disease; CHF, congestive heart failure; CRP, C-reactive protein; HDL-C, high-density lipoprotein cholesterol; MVPA, moderate-to-vigorous physical activity; PM, person-months of observation. ^a^ Unweighted sedentary behavior median was 486 min/day.

## Discussion

The purpose of this study was to examine the association between accelerometer-assessed sedentary behavior and residual-specific mortality. The motivation for this study was two-fold: (1) emerging work provides suggestive evidence of an association between sedentary behavior and all-cause mortality and cause-specific mortality for several major causes of death, but the relationship between sedentary behavior and residual-specific mortality has yet to be considered in the literature; and (2) a substantive proportion of deaths are attributable to residual-specific deaths. The main finding of this study was that sedentary behavior was independently associated with an increased hazard of residual-specific mortality. This association was somewhat modest, but appeared robust given the consistent association across the analytic models. An important finding, however, was the evidence of an interaction effect of sedentary behavior and total physical activity on residual-specific mortality.

These findings are in alignment with an accumulating body of research suggesting an independent association between sedentary behavior and all-cause and cause-specific mortality.^1-3,9-12^ Recently, Maher et al^13^ were the first to include ‘total physical activity’ as a covariate when examining the association between objectively-measured sedentary behavior and cardiometabolic-related health. Similar to the emerging work on this topic, they showed that, while adjusting for MVPA, sedentary behavior was detrimentally associated with 8 out of 11 evaluated cardiometabolic-related parameters. However, after adjusting for total physical activity, the associations between sedentary behavior and the cardiometabolic-related parameters effectively disappeared, with only 1 cardiometabolic-parameter (CRP) remaining statistically significant. In the present study, sedentary behavior remained statistically significantly associated with residual-specific mortality even after adjusting for total physical activity, but our observed interaction effect suggested that sedentary behavior was only statistically significantly associated with residual-specific mortality among those with below median total physical activity levels. Until future replicative work is done, it may be sensible to exercise caution when interpreting this interaction effect as there were few residual-specific deaths among those above the median total physical activity level.

Given that the specific causes of the residual mortalities were not identifiable, it is not fully possible to understand the mechanism behind the observed association between sedentary behavior and residual-specific mortality. When considering the NCHS codes for mortality, residual-specific mortality may include the following specific causes of death: (1) infectious and parasitic diseases, (2) in situ neoplasms, benign neoplasms and neoplasms of unknown behavior of specified and unspecified sites, (3) diseases of the blood and blood-forming organs and certain disorders involving the immune system, (4) endocrine, nutritional and metabolic disease, (5) mental and behavioral disorders, (6) Diseases of the nervous system, (7) diseases of the eye and adnexa, (8) diseases of the ear and mastoid process, (9) diseases of the circulatory system (other than heart disease), (10) diseases of the respiratory system, (11) diseases of the digestive system, (12) diseases of the skin and subcutaneous tissue, (13) diseases of the musculoskeletal and connective tissue, (14) diseases of the genitourinary system (other than nephritis, nephrotic syndrome and nephrosis), (15) pregnancy, childbirth and the puerperium, (16) certain conditions originating in the prenatal period, (17) congenital malformations, deformations and chromosomal abnormalities, (18) symptoms, signs and abnormal clinical and laboratory findings, not elsewhere defined, (19) external causes of mortality (other than accidents/unintentional injuries), and (20) factors influencing health status and contact with health services.

The potential underlying explanations for these residual deaths may include infection, immune dysfunction, systemic inflammation and mental dysfunction, of which have been shown to associate with sedentary behavior.^14,15^ Additional research is needed to confirm this potential pervasive effect of sedentary behavior on residual-specific mortality.

Limitations of this study include the relatively short follow-up period (80 months), relatively few residual-specific deaths observed (n = 144) and, like most observational studies, the assessment of the exposure variable occurring at only one time period. Notable strengths of this study include the objective measure of physical activity, national sample employed, and novel investigation.

In summary, sedentary behavior was associated with residual-specific mortality, with this observation occurring independent of MVPA and total physical activity. However, there was evidence to suggest that residual-specific mortality risk was a function of sedentary behavior and total physical activity. These findings highlight the need for future work to not only examine the association between sedentary behavior and health independent of total physical activity, but evaluate whether there is a joint effect of these two parameters on health. If future research does indeed confirm our findings, then intervention-based strategies at both the family and community levels will need to be put into place to not only promote physical activity, but also minimize prolonged sedentary.

## Acknowledgments

No funding was used to prepare this manuscript.

## Ethical approval

This study was approved by the ethics committee at the National Center for Health Statistics.

## Competing interests

We declare no conflicts of interest.

## Authors’ contributions

All authors were involved in the conceptualization of the study, revising the manuscript and interpreting the results. PDL computed the analyses and drafted the first draft of the manuscript.
